# MicroRNA-222 alleviates radiation-induced apoptosis by targeting BCL2L11 in cochlea hair cells

**DOI:** 10.1042/BSR20201397

**Published:** 2021-06-04

**Authors:** Yan-yan Zhang, Gao-yun Xiong, Xiao-xing Xie

**Affiliations:** Department of Otolaryngology, Tongde Hospital of Zhejiang Province, Hangzhou 310012, China

**Keywords:** BCL-2-like protein 11, cochlear cells, ionizing radiation, miR-222

## Abstract

Radiation-induced hair cell injury is detrimental for human health but the underlying mechanism is not clear. MicroRNAs (miRNAs) have critical roles in various types of cellular biological processes. The present study investigated the role of miR-222 in the regulation of ionizing radiation (IR)-induced cell injury in auditory cells and its underlying mechanism. Real-time PCR was performed to identify the expression profile of miR-222 in the cochlea hair cell line HEI-OC1 after IR exposure. miRNA mimics or inhibitor-mediated up- or down-regulation of indicated miRNA was applied to characterize the biological effects of miR-222 using MTT, apoptosis and DNA damage assay. Bioinformatics analyses and luciferase reporter assays were applied to identify an miRNA target gene. Our study confirmed that IR treatment significantly suppressed miR-222 levels in a dose-dependent manner. Up-regulation of miR-222 enhances cell viability and alleviated IR-induced apoptosis and DNA damage in HEI-OC1 cells. In addition, BCL-2-like protein 11 (BCL2L11) was validated as a direct target of miR-222. Overexpression of BCL2L11 abolished the protective effects of miR-222 in IR-treated HEI-OC1 cells. Moreover, miR-222 alleviated IR-induced apoptosis and DNA damage by directly targeting BCL2L11. The present study demonstrates that miR-222 exhibits protective effects against irradiation-induced cell injury by directly targeting BCL2L11 in cochlear cells.

## Introduction

Hearing impairment is detrimental for human health, affecting communication and impacting quality of life. The pathogenesis of hearing loss includes internal and external causes, such as mutations in deafness genes, aging, exposure to ototoxic compounds, exposure to excessive levels of sound, and irradiation therapy [1]. Particularly, hearing impairment induced by radiation therapy, such as sensorineural hearing loss (SNHL), is a common complication following radiotherapy [2,3]. Currently, no effective strategy has been developed for radiation-induced SNHL and the mechanisms underlying radiation-induced auditory cell death remain obscure.

MicroRNAs (miRNAs) are a group of small, noncoding RNAs. By post-transcriptional regulation of target mRNAs through complementary binding to the 3′ untranslated region (UTR), they play diverse roles in cellular behaviors, including cell proliferation, apoptosis, differentiation, and angiogenesis [4]. Most studies mainly focus on the relationship between and miRNAs and radiotherapy-induced cellular responses in cancers [5]. Moreover, the specific role of miRNAs in radiation-induced cochlea hair cell injury is limited [6]. For example, Zhou et al. established a gentamicin-induced cochlear injury mouse model and revealed the repressing role of Notch signaling pathway during the regeneration of hair cells; moreover, they demonstrated that miR-183 is implicated in the regeneration of hair cells via Notch signaling pathway [7]. Another study investigated the functions of miR-207 in the regulation of ionizing radiation (IR)-induced cell death in auditory cells; their findings suggested that miR-207 promoted radiation-induced apoptosis by directly targeting Akt3 in cochlea hair cells [8].

Accumulating evidence suggests that miR-222, by regulating different genes at the post-transcriptional level, has important roles in various types of cellular biological processes, including responses to IR [9,10]. However, the functions of miR-222 in the IR-induced hair cell injury have not been elucidated. Thus, our present study aimed to investigate the expression profile of miR-222 in the auditory cell line HEI-OC1 in response to IR exposure. Further investigation revealed that miR-222 exerted its biological role by negatively regulating BCL-2-like protein 11 (BCL2L11) as a direct target. Therefore, the present study provides a novel mechanism for IR-induced apoptosis in cochlea hair cells.

## Materials and methods

### Cell culture

The HEI-OC1 cells were obtained from the Shanghai Institute of Biological Sciences, Chinese Academy of Sciences (Shanghai, China). Cells were maintained in Dulbecco’s modified Eagle’s medium (DMEM) (Gibco, Grand Island, NY, U.S.A.) containing 10% fetal bovine serum (FBS) (Gibco, Grand Island, NY, U.S.A.). Cells were cultured at 33°C in a 10% CO_2_ humidity-controlled incubator.

### Irradiation

The HEI-OC1 cells were irradiated at a distance of 100 cm from the source to the axis using a 6-MV linear accelerator (LINAC; 2300EX; Varian Co., Palo Alto, CA, U.S.A.) at a dose rate of 5.0 Gy/min. All the irradiations were performed at room temperature. After irradiation, the culture plates were returned to an incubator under the same conditions as previously described.

### Cell viability assay

For determination of cell viability, MTT assay was performed based on the manufacturer’s protocol (Sigma, St. Louis, MO, U.S.A.). In brief, the HEI-OC1 cells were seeded on to 96-well plates and transfected with mimic control, miR-222 mimic, inhibitor control, and miR-222 inhibitor followed by radiation treatment. The cell viability in each well was determined by adding 20 μl of 5 mg/ml MTT (Sigma, St. Louis, MO, U.S.A.) solution followed by culture for an additional 2 h. The optical absorbance was measured using an ELISA reader at a wavelength of 490 nm. Each experiment was performed in triplicate.

### Transfection

Mimic control, miR-222 mimic, inhibitor control, and miR-222 inhibitor, BCL2L11 plasmids, and control were purchased from RiboBio (Guangzhou, Guangdong, China). The BCL2L11 overexpression was conducted by constructing the full-length BCL2L11 sequences into cells via pEX-2 plasmids (GenePharma, Shanghai, China). After the cells were seeded in six-well plates, transfection was performed using Lipofectamine 2000 (Invitrogen, Carlsbad, CA, U.S.A.) according to the manufacturer’s instructions.

### Apoptosis assay

Annexin V-FITC/PI staining was performed to quantify the percentage of apoptotic cells. The cells were stained using the Annexin V-FITC Apoptosis Detection kit (Invitrogen, Inc., Carlsbad, CA, U.S.A.) according to the manufacturer’s protocol. After being washed with PBS, the HEI-OC1 cells were resuspended in binding buffer, incubated with Annexin V-FITC and PI, and subject to flow cytometry. The results were analyzed using the BD AccuriC6 Software (BD Biosciences).

### Real-time PCR

Total RNAs were extracted from cultured cells using TRIzol reagent (TaKaRa, Dalian, China) and reversely transcribed into cDNA using a PrimeScript RT Master Mix Perfect Real Time (TaKaRa, Dalian, China) kit. Real time PCR was performed using an ABI 7900 system. The transcript levels of miR-222 and BCL2L11 were determined using the equation 2^−ΔΔ*C*_t_^, with the small noncoding RNA U6 and housekeeping gene *β-actin* as internal controls, respectively. The primers used were: miR-222 forward, 5′-AGCTACATCTGGCTACTGG-3′, and reverse, 5′-GTATCCAGTGCAGGGTCC-3′; U6 forward, 5′-CTCGCTTCGGCAGCACA-3′ and reverse, 5′-TGGTGTCGTGGAGTCG-3′. BCL2L11 forward 5′- TAAGTTCTGAGTGTGA CCGAGA-3′ and reverse, 5′- CCCTGTTGCTGTAGCCAAATTC-3′. β-actin forward, 5′-TTGCCGACAGGATGCAGAAGGA-3′ and reverse, 5′-AGGTGGACAGCGAGGCCAG GAT-3′. The assays were performed in triplicate for each case.

### Immunofluorescent staining for γ-H2AX

The DNA damage was evaluated by performing immunofluorescent staining for γ-H2AX. The HEI-OC1 cells were grown on polylysine-coated cover glasses. After radiation, the cells were fixed in 4% paraformaldehyde, permeabilized in Triton X-100 and blocked in 1% goat serum. Subsequently, the cells were incubated with the primary antibody against γ-H2AX (1:100 dilution; Abcam, San Francisco, CA, U.S.A.) overnight at 4°C, followed by incubation with Alexa Flour 488 secondary antibody (1:200 dilution; Abcam, San Francisco, CA, U.S.A.) for 1 h at room temperature in the dark. After being washed three times in PBS, cells were stained with DAPI and observed using a fluorescence microscope (Olympus, Tokyo, Japan).

### Bioinformatics analyses

The mature sequence of miR-222 was obtained from the miRNA database (http://www.mirbase.org/). miRanda (http://www.microrna.org), PicTar (http://pictar.mdc-berlin.de/) and TargetScan (http://www.targetscan.org/) were used to predict the target gene of miR-222.

### Luciferase assay

Luciferase reporter assay was performed by co-transfection of pLUC-REPORT vector (Ambion, Austin, TX, U.S.A.) containing wildtype or mutant 3′UTR of BCL2L11 containing the putative miR-222 binding site into HEK293T cells in 96-well plates using Lipofectamine 2000 (Invitrogen, Carlsbad, CA, U.S.A.) according to the manufacturer’s instructions. For luciferase reporter assay, 48 h after transfection, luciferase activity was measured using the Dual-Luciferase Reporter Assay System (Promega, Madison, WI, U.S.A.). Luciferase activity was read by SpectraMax M5 (Molecular Devices, Sunnyvale, CA, U.S.A.). Experiments were performed in triplicate and repeated three times.

### Western blot

HEI-OC1 cells were washed and suspended in lysis buffer (Sigma–Aldrich, St. Louis, MO, U.S.A.). Equal amounts of protein samples were separated by SDS/PAGE and transferred to polyvinylidene difluoride membranes (Millipore Corp., Bedford, MA, U.S.A.). After being blocked in PBS containing 5% powdered milk, the membranes were incubated with primary antibodies against BCL2L11 (1:1000, dilution; Abcam, San Francisco, CA, U.S.A.) and β-actin (1:2000, dilution; Abcam, San Francisco, CA, U.S.A.) overnight. Then, the membrane was incubated with appropriate horseradish peroxidase–conjugated secondary antibodies at room temperature for 1 h. Protein bands were detected using Super Signal Enhanced Chemiluminescence kit (Pierce, Rockford, IL, U.S.A.).

### Statistical analysis

Data are expressed as mean ± standard deviation (SD). Comparison between groups was performed using the Student’s *t* test or one-way ANOVA. Values of *P*<0.05 were considered statistically significant.

## Results

### MiR-222 expression is suppressed by IR and promotes cell growth

**Figure 1:**
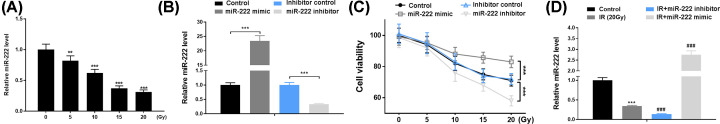
miR-222 expression is suppressed by IR and promotes cell growth (**A**) Real-time PCR was performed to detect the expression of miR-222 in HEI-OC1 cells following at irradiation at 5, 10, 15 and 20 Gy. (**B**) HEI-OC1 cells were transfected with mimic control, miR-222 mimic, inhibitor control and miR-222 inhibitor independently. Real-time PCR was performed to detect the level of miR-222 at 48 h after transfection. (**C**) After transfection, HEI-OC1 cells were subjected to the MTT assay at 24 h after IR (5, 10, 15, 20 Gy) or without IR. (**D**) Real-time PCR was performed to detect the level of miR-222 at 24 h after IR (20 Gy) or without IR. **P<0.01, ***P<0.001 vs. control; ^###^*P*<0.001 vs. IR group.

Firstly, HEI-OC1 cells were exposed to irradiation at irradiation at 5, 10, 15 and 20 Gy. The real-time PCR results confirmed that miR-222 was significantly down-regulated after IR in a dose-dependent manner (Figure 1A). After we successfully transfected miR-222 mimics or miR-222 inhibitor into HEI-OC1 cells (Figure 1B), MTT assay was performed to evaluate the role of miR-222 in HEI-OC1 cells. Consequently, we found that overexpression of miR-222 significantly enhanced cell growth in HEI-OC1 cells after IR at 5, 10, 15 and 20 Gy, but not in cells without IR. However, down-regulation of miR-222 significantly inhibited cell growth in HEI-OC1 cells after IR at 5, 10, 15 and 20 Gy, but not in cells without IR (Figure 1C). Additionally, the real-time PCR results revealed that miR-222 was significantly down-regulated after IR, but was dramatically elevated following transfection with miR-222 mimic (Figure 1D). Collectively, these findings confirm that miR-222 could affect cell growth in HEI-OC1 cells exposed to irradiation.

### MiR-222 inhibits IR-induced apoptosis and DNA damage in HEI-OC1 cells

**Figure 2:**
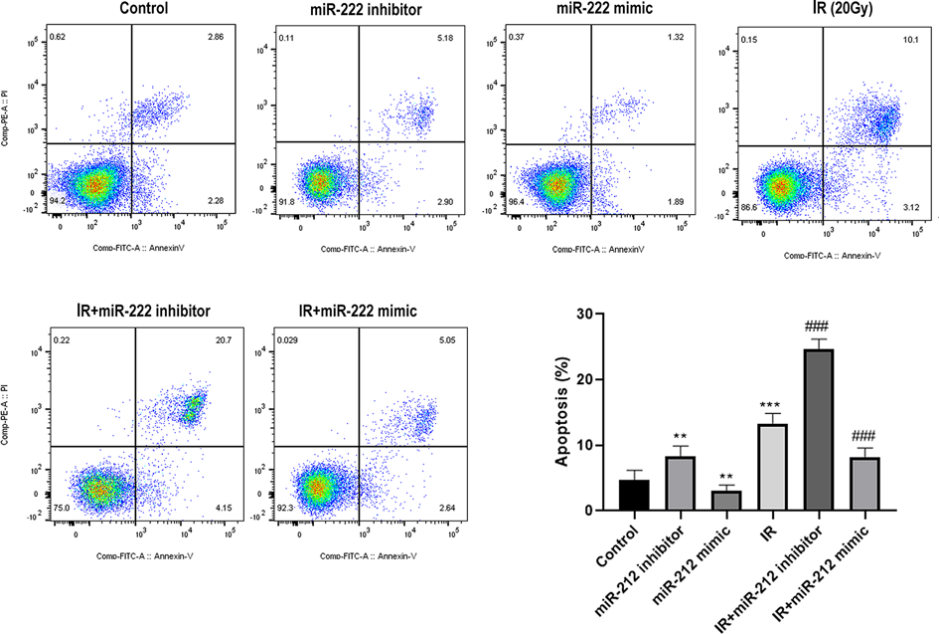
miR-222 inhibits IR-induced apoptosis HEI-OC1 cells were transfected with mimic control, miR-222 mimic, inhibitor control and miR-222 inhibitor independently. The apoptosis assay using flow cytometry was performed 24 h after IR (5, 10, 15, 20 Gy) or without IR. **P<0.01, ***P<0.001 vs. control; ^###^*P*<0.001 vs. IR group.

**Figure 3:**
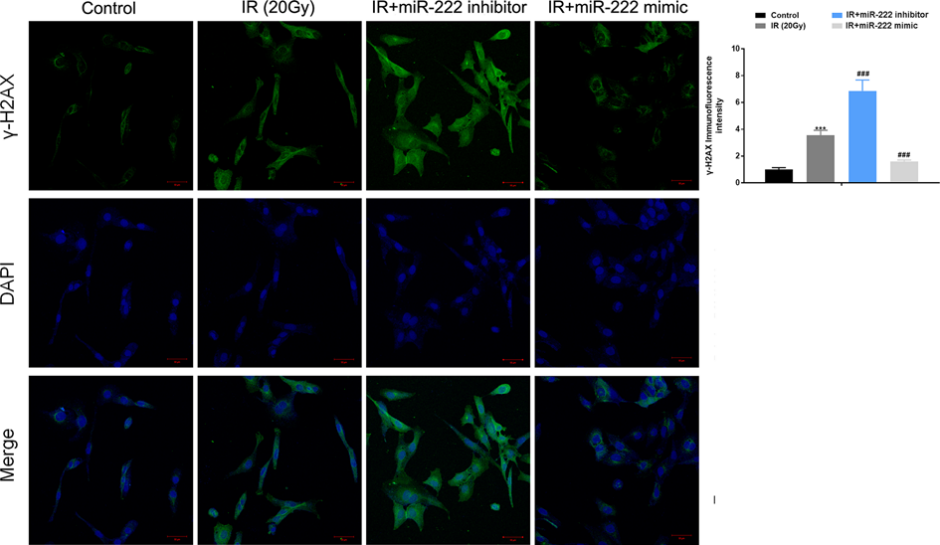
miR-222 inhibits IR-induced DNA damage HEI-OC1 cells were transfected with mimic control, miR-222 mimic, inhibitor control and miR-222 inhibitor independently. Immunofluorescent staining for γ-H2AX was performed at 24 h after 20 Gy IR of transfected cells. ***P<0.001 vs. control; ^###^*P*<0.001 vs. IR group.

Furthermore, we investigated whether miR-222 affected apoptosis in HEI-OC1 cells. The HEI-OC1 cells were transfected with mimic control, miR-222 mimic, inhibitor control, and miR-222 inhibitor with or without irradiation at 20 Gy. The flow cytometry results indicated that down-regulation of miR-222 significantly enhanced apoptosis while overexpression of miR-222 suppressed apoptosis in cells without irradiation (Figure 2). Moreover, in irradiated cells, miR-222 knockdown further elevated IR-induced apoptosis, whereas overexpression of miR-222 significantly suppressed IR-induced apoptosis in HEI-OC1 cells (Figure 2). In addition, we investigated whether miR-222 affected DNA damage in HEI-OC1 cells. As expected, irradiation at 20 Gy significantly induced DNA damage. However, immunofluorescent staining for γ-H2AX showed that transfection with miR-222 resulted in lower levels of γ-H2AX foci, whereas transfection with miR-222 inhibitors resulted in significantly higher γ-H2AX levels in HEI-OC1 cells after IR at 20 Gy (Figure 3). Taken together, these results suggest that miR-222 could inhibit IR-induced DNA damage in HEI-OC1 cells.

### BCL2L11 is a direct target of miR-222

**Figure 4:**
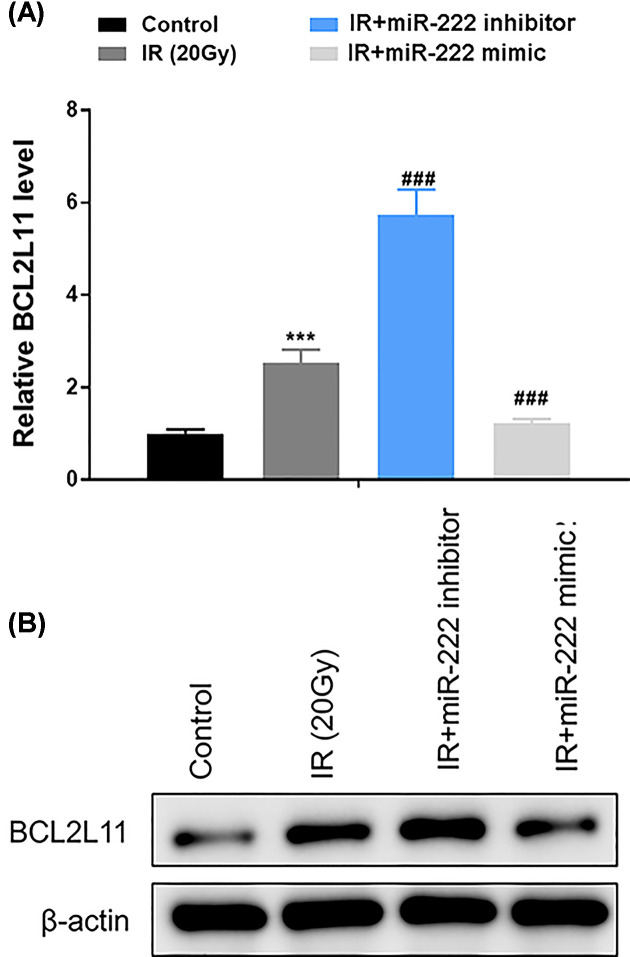
miR-222 negatively regulates BCL2L11 expression HEI-OC1 cells were transfected with mimic control, miR-222 mimic, inhibitor control and miR-222 inhibitor independently. Real-time PCR (**A**) and Western blot (**B**) were performed at 24 h after 20 Gy IR of transfected cells to detect the mRNA and protein expression of BCL2L11. ***P<0.001 vs. control; ^###^*P*<0.001 vs. IR group.

**Figure 5:**
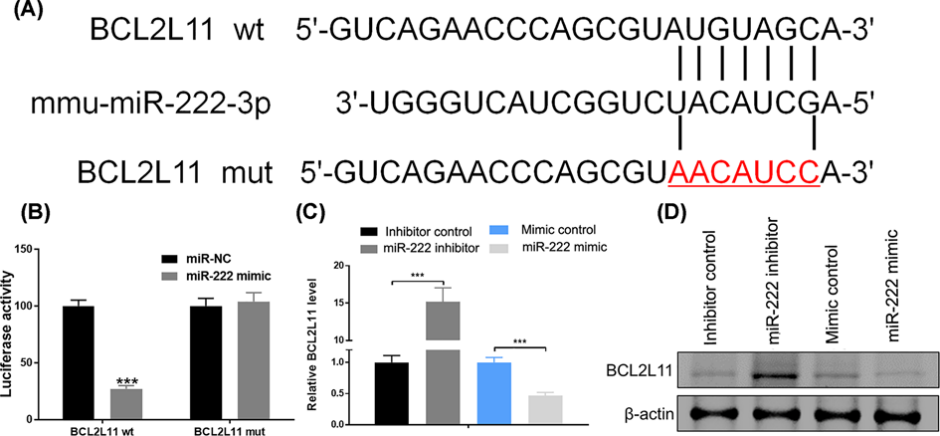
BCL2L11 is a direct target of miR-222 (**A**) The structure of BCL2L11 3′UTR complementary to the seed sequence of miR-222 and the mutant sequence. (**B**) The relative luciferase activity in HEI-OC1 cells was determined after co-transfection with BCL2L11 3′UTR or mut-BCL2L11 3′UTR plasmids and miR-222 or control. (**C**) BCL2L11 mRNA expression was determined using real-time PCR after transfection with miR-222 mimic or miR-222 inhibitor. (**D**) Expression levels of BCL2L11 were determined by Western blot in miR-222 mimic or miR-222 inhibitor transfected cells. ***P<0.001 vs. control.

Next, the real-time PCR results revealed that IR induced elevation of BCL2L11 in HEI-OC1 cells. Such increase in BCL2L11 by IR was further enhanced in HEI-OC1 cells transfected miR-222 inhibitor. By contrast, up-regulation of miR-222 significantly suppressed the mRNA expression of BCL2L11 in IR-treated cells (Figure 4A). Consistently, Western blot analysis showed that up-regulation of miR-222 inhibited protein expression of BCL2L11, whereas down-regulation of miR-222 enhanced BCL2L11 in IR-treated HEI-OC1 cells (Figure 4B). Next, we identified whether BCL2L11 is a target for miR-222. miRNA target prediction programs, including miRanda, PicTar and TargetScan, showed that the 3′UTR of *BCL2L11* gene contained the conserved binding sites for miR-222 (Figure 5A). To validate that BCL2L11 is a target of miR-222, luciferase reporters that contained the 3′UTR or a mutated sequence within the biding site of BCL2L11 gene were generated. Consequently, the activity of wildtype BCL2L11-3′UTR was significantly decreased in cells co-transfected with miR-222 and wildtype BCL2L11, but remained unchanged in cells transfected with the mutated BCL2L11 combined with miR-222 (Figure 5B). Moreover, the mRNA and protein levels of BCL2L11 was suppressed following treatment with miR-222 mimics, but elevated after addition with miR-222 inhibitor, as shown by real-time PCR and Western blot, respectively (Figure 5C,D). Additionally, the interferon-regulatory factor 2 (IRF2) was validated to be another target of miR-222. However, functional assays revealed that IRF2 was not involved in the protective role of miR-222 in irradiated HEI-OC1 cells (Supplementary Figure S1). Collectively, these findings showed that BCL2L11 was a direct target gene of miR-222 in HEI-OC1 cells.

### Up-regulation of BCL2L11 mimics the effects of miR-222

**Figure 6:**
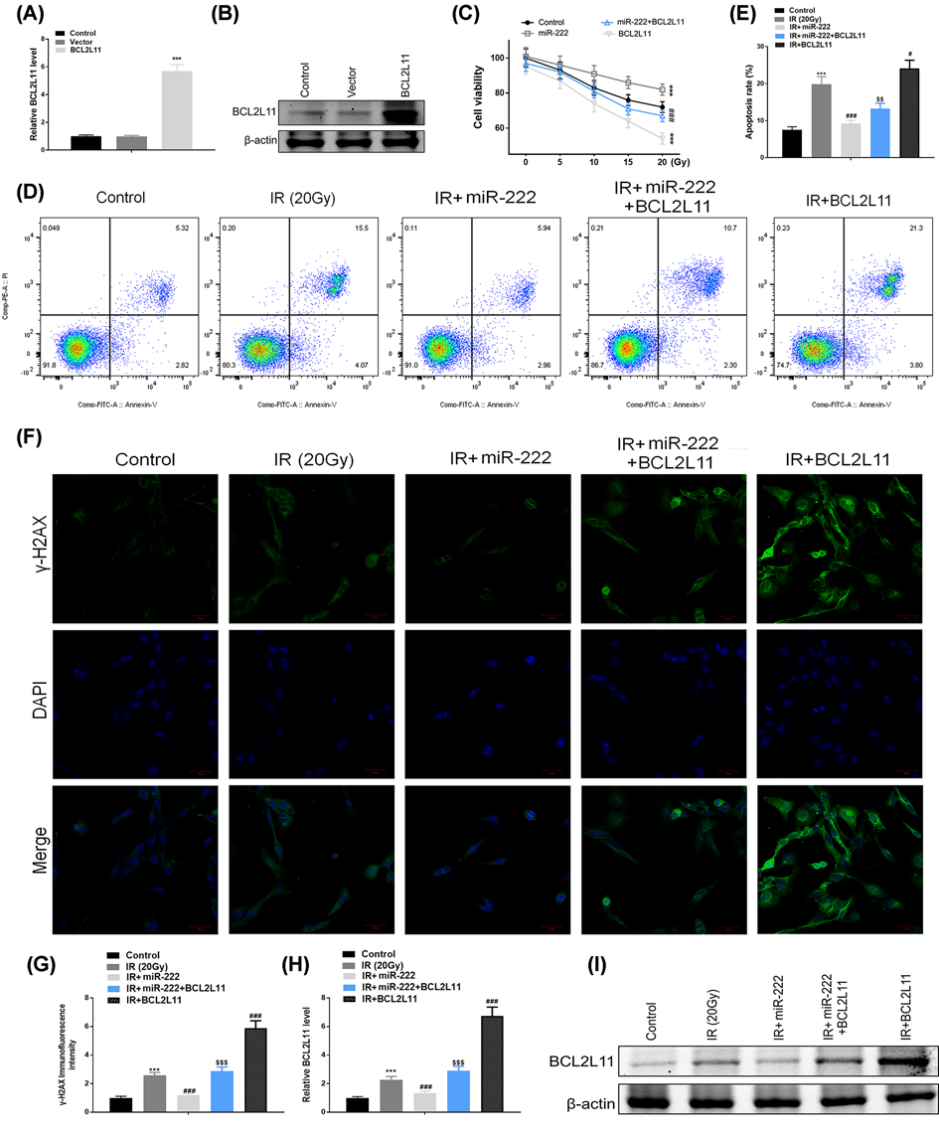
Up-regulation of BCL2L11 mimics the effects of miR-222 Expression levels of BCL2L11 were determined using real-time PCR (**A**) and Western blot (**B**) analyses in HEI-OC1 cells transfected with BCL2L11 plasmids or control. (**C**) HEI-OC1 cells were transfected with miR-222 or BCL2L11 plasmids alone or in combination. HEI-OC1 cells were subjected to the MTT assay at 24 h after IR (5, 10, 15, 20 Gy) or without IR. (**D**,**E**) After transfection, the apoptosis was determined using flow cytometry performed 24 h after irradiation with 20 Gy. (**F**,**G**) Expression of γ-H2AX was determined by immunofluorescent staining in transfected cells 24 h after irradiation with 20 Gy. (**H**,**I**) The mRNA and protein expression levels of BCL2L11 were determined at 24 h after IR in transfected cells. ***P<0.001 vs. control; ^#^*P*<0.05,^ ###^*P*<0.001 vs. IR group; ^$$^*P*<0.01, ^$$$^*P*<0.001 vs. IR+pCDH-miR-222.

We next examined whether up-regulation of BCL2L11 exerted similar effects of miR-222 in HEI-OC1 cells. Firstly, we successfully enhanced the mRNA and protein levels of BCL2L11 protein (Figure 6A,B). MTT assay was performed to evaluate the role of BCL2L11 in miR-222 mimic-transfected HEI-OC1 cells. Overexpression of BCL2L11 remarkably suppressed HEI-OC1 cell viability after IR at 5, 10, 15, and 20 Gy. Moreover, up-regulation of miR-222 significantly enhanced cell growth, which was reversed in HEI-OC1 cells co-transfected with miR-222 mimic and BCL2L11 plasmids (Figure 6C). In addition, apoptosis analysis showed that HEI-OC1 cells transfected with BCL2L11 exhibited greater apoptosis compared with control, whereas miR-222 transfection suppressed apoptosis. However, the suppression of IR-induced apoptosis by miR-222 was partly abolished in HEI-OC1 cells co-transfected with miR-222 mimic and BCL2L11 plasmids (Figure 6D,E). In addition, we investigated whether miR-222 exerted its suppressive role on DNA damage in a BCL2L11-dependent manner. As expected, HEI-OC1 cells transfected with BCL2L11 exhibited significantly enhanced DNA damage compared with control, but transfection with miR-222 could alleviate IR-induced DNA damage. Nevertheless, the suppression of IR-induced DNA damage by miR-222 was partly reversed in cells co-transfected with BCL2L11 (Figure 6F,G). Additionally, the real-time PCR and Western blot results revealed that BCL2L11 was significantly elevated following IR, and such increase was even more obvious after transfection with BCL2L11 plasmids. By contrast, IR-induced up-regulation of BCL2L11 was dramatically inhibited by co-transfection with miR-222 mimic (Figure 6H,I). Taken together, these results suggest that miR-222 prevents against IR-induced cell injury via targeting BCL2L11 in HEI-OC1 cells.

## Discussion

Exposure to radiotherapy is a critical cause for inner-ear damage in 20–50% of patients [11]. miRNAs are important factors for post-transcriptional process that controls gene expression at mRNA level. Accumulating evidence support the critical roles of miRNAs in development and progression of hearing loss [12,13]. In the present study, IR exposure led to a dose-dependent up-regulation of miR-222 in HEI-OC1 cells, suggesting that miR-222 may be involved in auditory pathway.

To the best of our knowledge, miR-222 has not been thoroughly investigated. Most studies mainly focus on the relationship between and miR-222 and radiotherapy-induced cellular responses in cancers. Previously, miR-222 was reported to be up-regulated in response to IR; moreover, overexpression of miR-222 enhanced radiosensitivity by targeting CD47-ERK pathway in cancer cells [14]. A recent study suggested that miR-222 was up-regulated in nasopharyngeal carcinoma (NPC) tissues and malignant cell lines compared with adjacent normal samples and cell lines; moreover, miR-222 up-regulation significantly increased cell growth and conferred radioresistance by targeting phosphatase and tensin homolog (PTEN) in NPC cells [15]. The different roles of miR-222 may be dependent on the cellular context and its target genes. However, the function of miR-222 in cochlea hair cells has not been investigated. Our study found that miR-222 was significantly down-regulated after IR in a dose-dependent manner. In addition, overexpression of miR-222 enhanced cell growth and alleviated IR-induced apoptosis in HEI-OC1 cells. By contrast, down-regulation of miR-222 dramatically inhibited cell growth and enhanced apoptosis in irradiated cells.

It is well known that DNA is the major target of radiation effects. The unsuccessful repair of double-strand break (DSBs) may result in lethal consequences, such as apoptosis. H2AX phosphorylation is an early step in the response to DNA damage, and γ-H2AX has been verified to be one of the earliest markers of DSBs after IR [16,17]. In the present study, irradiation exposure remarkably induced γ-H2AX expression and DNA damage. However, overexpression of miR-222 resulted in lower levels of γ-H2AX foci in IR-treated cells. These findings indicated that miR-222 suppressed IR-induced DNA damage and thus alleviated apoptosis in IR-treated cells. Collectively, these findings confirm that miR-222 could protect against IR-induced cell injury by promoting cell viability and suppressing DNA damage and apoptosis in HEI-OC1 cells exposed to irradiation.

BCL2L11 is a member of BCL-2 family and is located in the outer membrane of mitochondria, where this protein plays crucial roles in mediating excitotoxic apoptosis, apoptosis-inducing factor translocation and mitochondrial depolarization [18]. In our study, BCL2L11 has been verified to be a direct target of miR-222. Recent studies have reported that BCL2L11 regulates many biological processes, including cell growth, apoptosis, migration, autophagy and cellular response to IR [19,20]. However, the detailed information that BCL2L11 regulates cell growth and apoptosis in cochlea hair cells remains unknown. In our study, BCL2L11 was found to be induced by IR exposure, and specifically inhibited by miR-222, whereas down-regulation of miR-222 enhanced BCL2L11 in HEI-OC1 cells treated with or without IR, suggesting that BCL2L11 was a direct target of miR-222. In addition, overexpression of BCL2L11 resulted in a decrease in cell viability, an increase in apoptosis and DNA damage in irradiated HEI-OC1 cells. Moreover, the protective effects of miR-222 were partly abolished by up-regulation of BCL2L11 in terms of cell viability, apoptosis and DNA damage in IR-treated HEI-OC1 cells.

As previously described, the therapeutic strategy against IR-induced cochlea hair cell death are limited. Therefore, identification of novel therapeutic agents is critical. Among the regulators of protein-coding genes, miRNA seems to be an ideal choice. In our study, specific up-regulation of miR-222 exhibits protective effects against irradiation-induced cell injury by directly targeting BCL2L11 in cochlear cells. Thus, further studies are necessary to confirm such effects of miR-222 *in vivo*.

In conclusion, our study firstly demonstrates that overexpression of miR-222 suppresses IR-induced apoptosis and DNA damage by directly targeting BCL2L11 in irradiated auditory cells. These findings provide a novel mechanism for IR-induced apoptosis in cochlea hair cells, contributing to the development of potential protectants for IR-induced hearing impairment.

## Supplementary Material

Supplementary Figure S1-S9

## Data Availability

The data that support the findings of the present study are available from the corresponding author upon reasonable request.

## Abbreviations

BCL2L11: BCL-2-like protein 11
DSB: double-strand break
IR: ionizing radiation
IRF2: interferon-regulatory factor 2
miRNA: microRNA
NPC: nasopharyngeal carcinoma
SNHL: sensorineural hearing loss
UTR: untranslated region
