# Supplementary material for: MicroRNA-222 alleviates radiation-induced apoptosis by targeting BCL2L11 in cochlea hair cells
Source: Biosci Rep. 2021 Jun 4;41(6):BSR20201397. doi: 10.1042/BSR20201397 (PMC8182987; doi:10.1042/BSR20201397)
Supplement: Supplementary Figure S1-S9 [file BSR-2020-1397_supp.pdf]

Supplementary figure

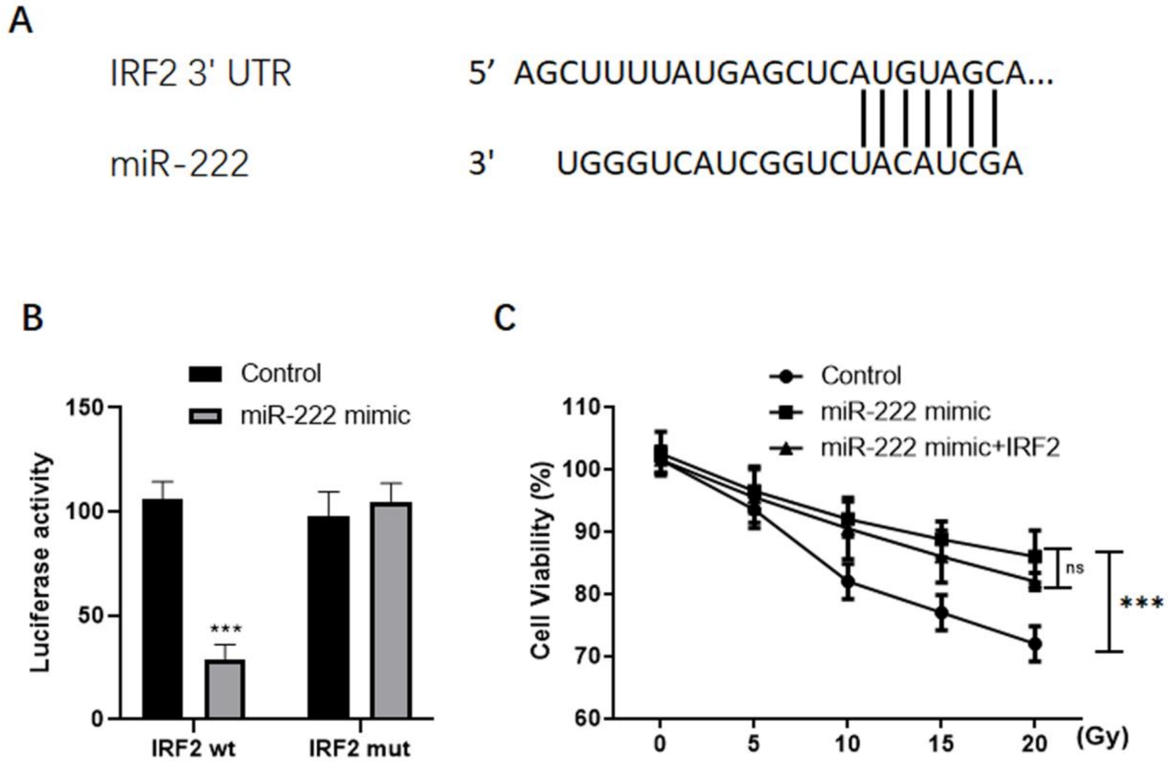

Figure S1 IRF2 is a target of miR-222 but not involved in its functional relevance in HEI-OC1 cells.

(A) The structure of IRF2 3′-UTR complementary to the seed sequence of miR-222. (B) The relative luciferase activity in HEI-OC1 cells was determined after co-transfection with IRF2 3′-UTR or mut-IRF2 3′-UTR plasmids and miR-222 or control. (C) HEI-OC1 cells were subjected to the MTT assay after IR (5, 10, 15, 20 Gy). *** P< 0.001; ns, not significant.

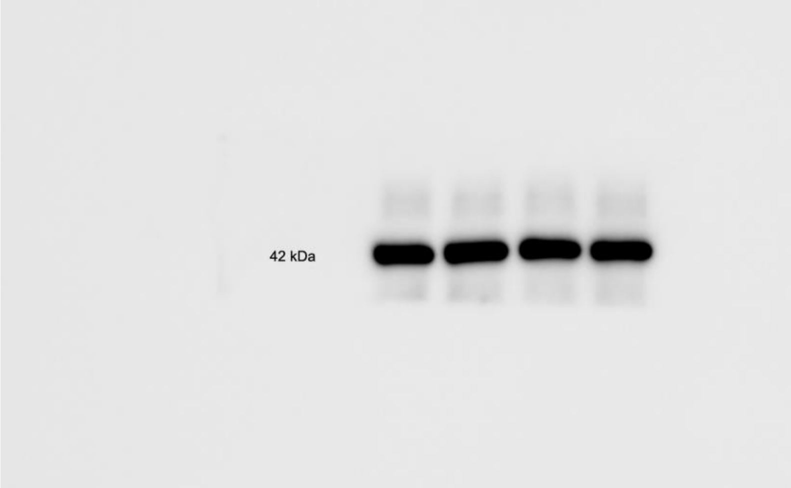

Figure S2 original blot-Figure 4B β-actin.

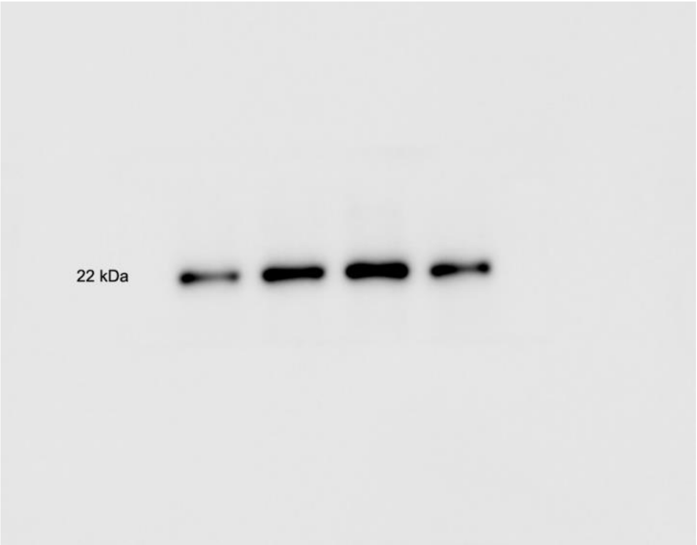

Figure S3 original blot-Figure 4B BCL2L11.

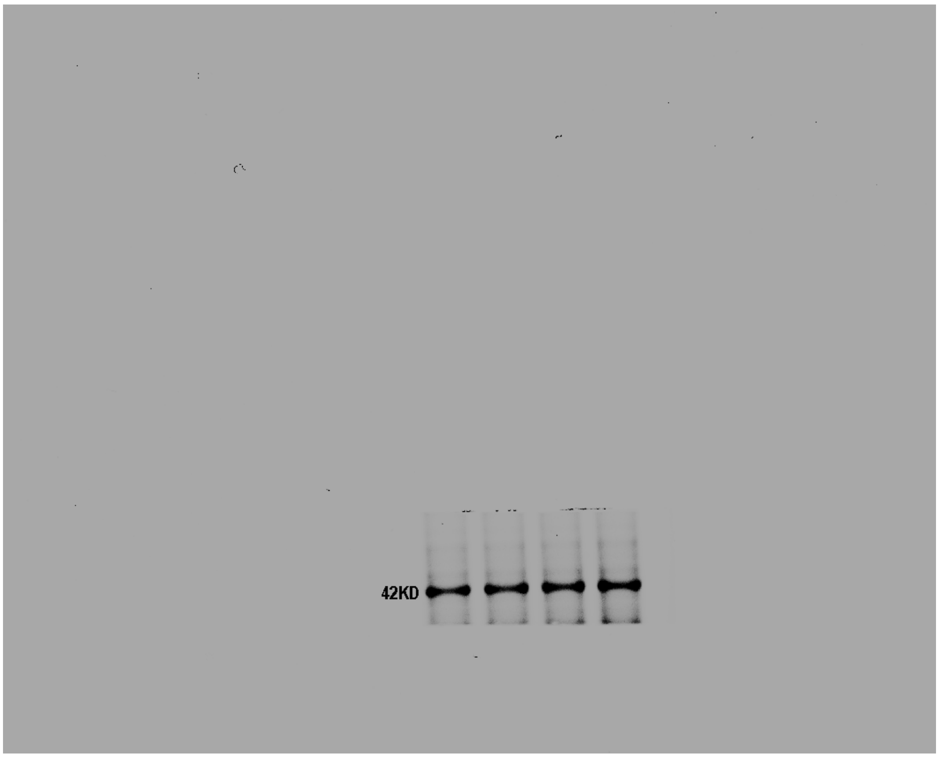

Figure S4 original blot-Figure 5D β-actin.

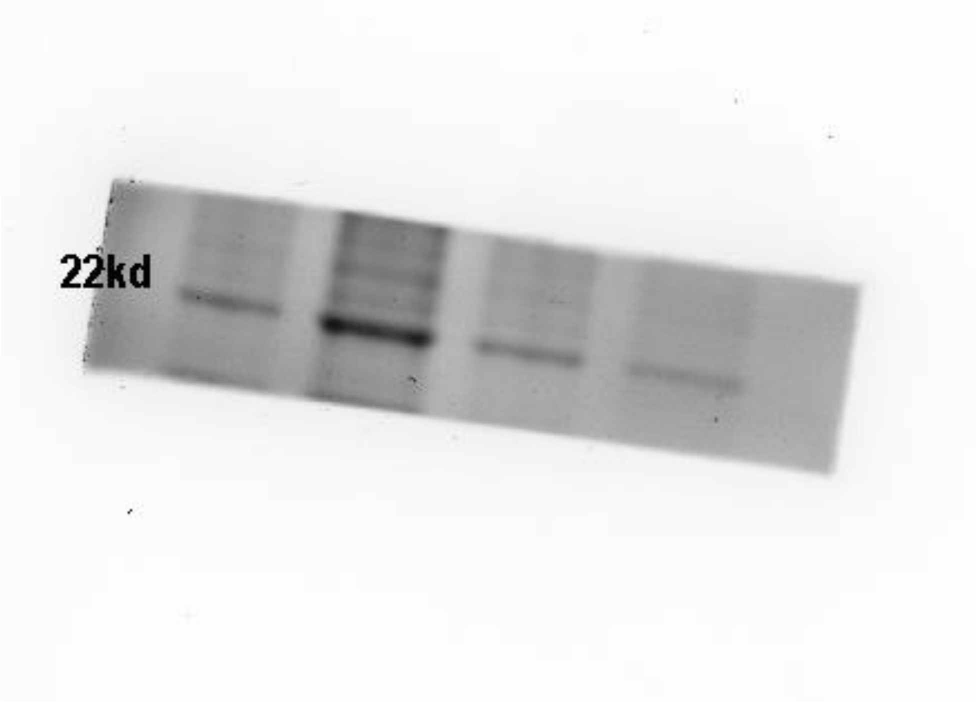

Figure S5 original blot-Figure 5D BCL2L11.

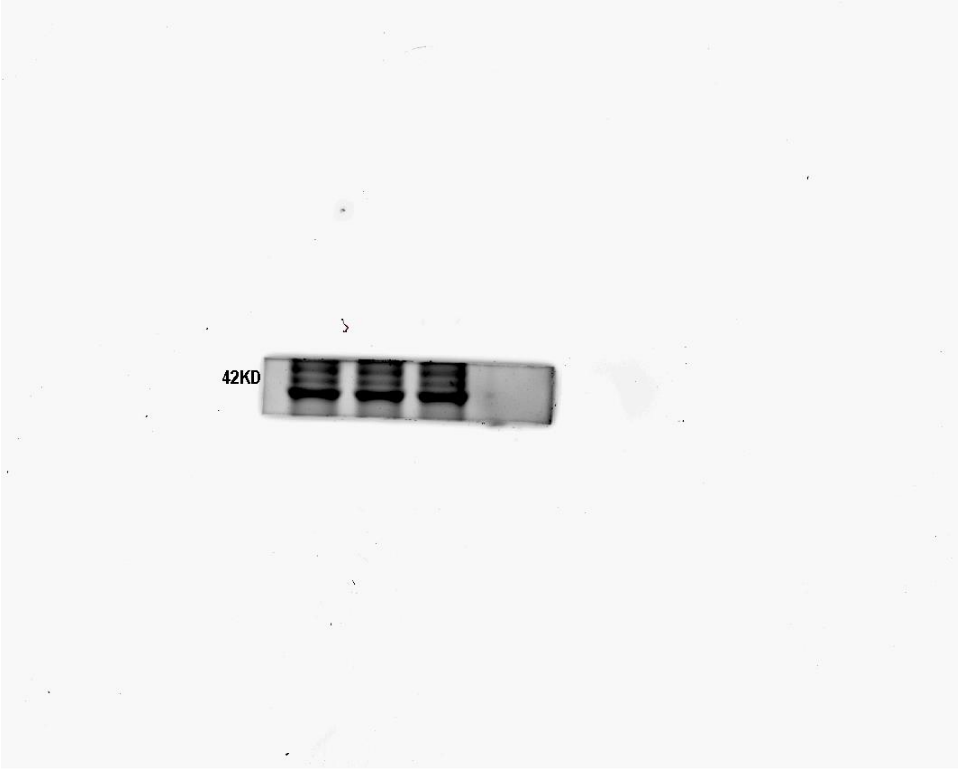

Figure S6 original blot-Figure 6B β-actin.

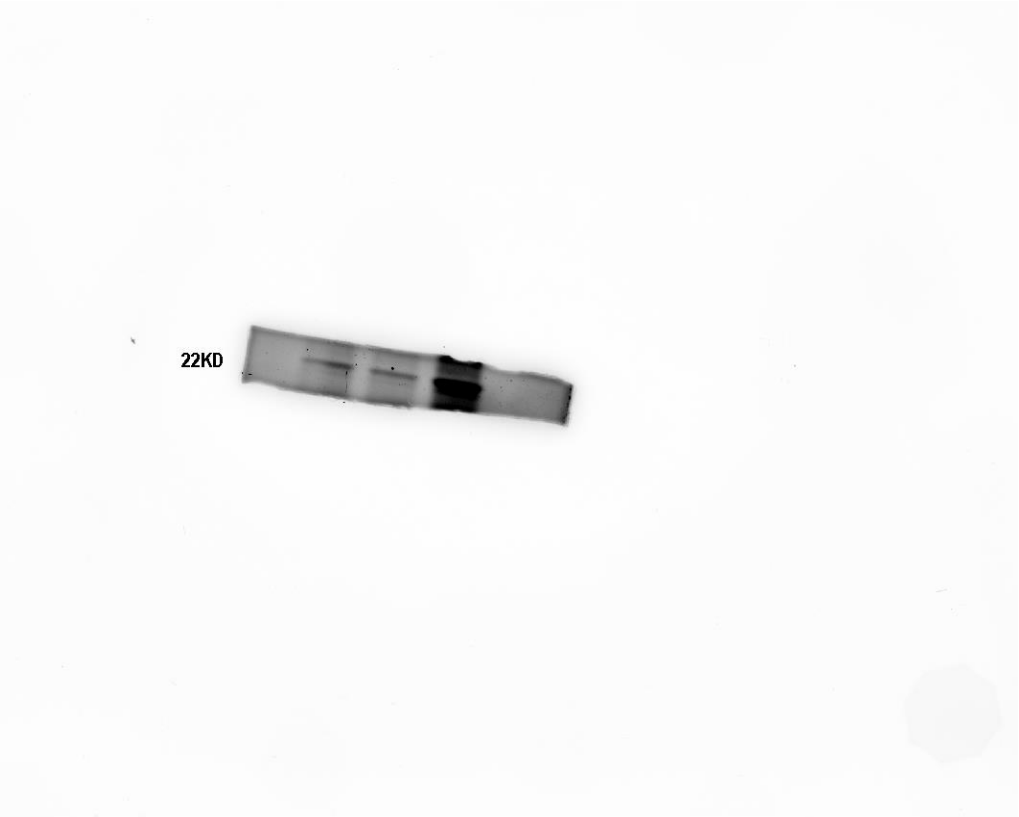

Figure S7 original blot-Figure 6B BCL2L11.

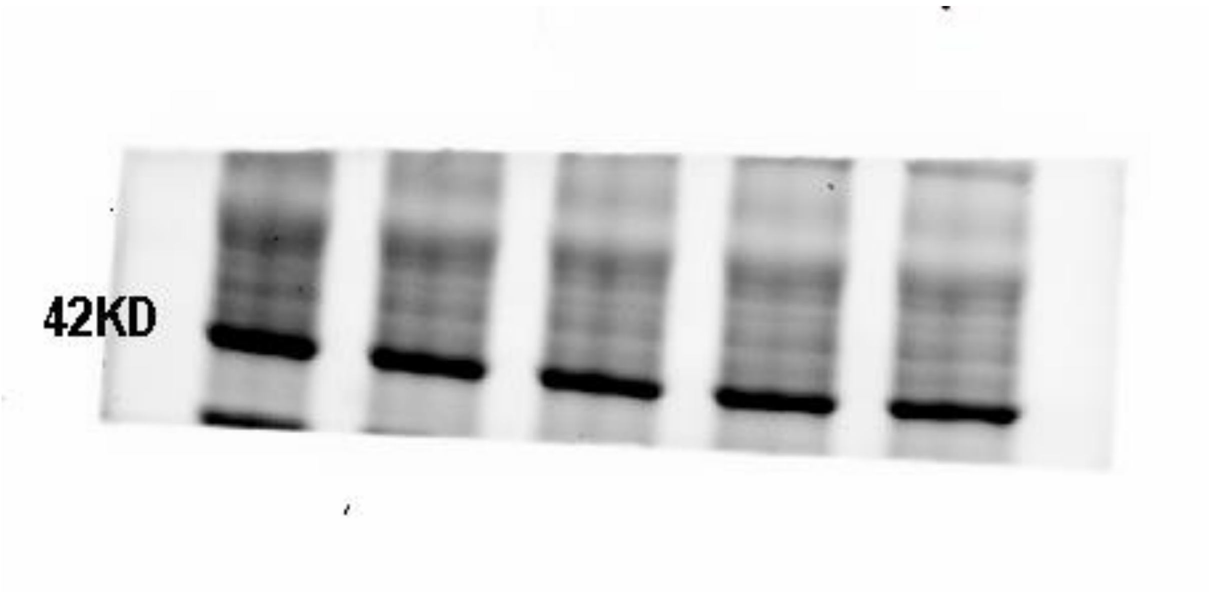

Figure S8 original blot-Figure 6I β-actin.

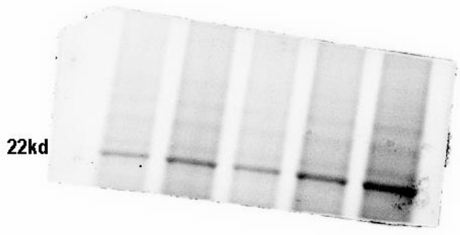

Figure S9 original blot-Figure 6I BCL2L11.
